# Cross-cultural validation of the revised Green et al., paranoid thoughts scale

**DOI:** 10.1017/S0033291724000072

**Published:** 2024-07

**Authors:** Björn Schlier, Tania M. Lincoln, Jessica L. Kingston, Suzanne H. So, Brandon A. Gaudiano, Eric M. J. Morris, Lyn Ellett

**Affiliations:** 1 Universität Hamburg, Hamburg, Germany; 2 University of Wuppertal, Wuppertal, Germany; 3 Royal Holloway, University of London, London, UK; 4 The Chinese University of Hong Kong, Hong Kong SAR; 5 Brown University, Providence, RI, USA; 6 La Trobe University, Melbourne, Australia; 7 University of Southampton, Southampton, UK

**Keywords:** general population, measurement invariance, paranoia, persecutory delusions

## Abstract

**Background:**

With efforts increasing worldwide to understand and treat paranoia, there is a pressing need for cross-culturally valid assessments of paranoid beliefs. The recently developed Revised Green et al., Paranoid Thoughts Scale (R-GPTS) constitutes an easy to administer self-report assessment of mild ideas of reference and more severe persecutory thoughts. Moreover, it comes with clinical cut-offs for increased usability in research and clinical practice. With multiple translations of the R-GPTS already available and in use, a formal test of its measurement invariance is now needed.

**Methods:**

Using data from a multinational cross-sectional online survey in the UK, USA, Australia, Germany, and Hong Kong (*N* = 2510), we performed confirmatory factory analyses on the R-GPTS and tested for measurement invariance across sites.

**Results:**

We found sufficient fit for the two-factor structure (ideas of reference, persecutory thoughts) of the R-GPTS across cultures. Measurement invariance was found for the persecutory thoughts subscale, indicating that it does measure the same construct across the tested samples in the same way. For ideas of reference, we found no scalar invariance, which was traced back to (mostly higher) item intercepts in the Hong Kong sample.

**Conclusion:**

We found sufficient invariance for the persecutory thoughts scale, which is of substantial practical importance, as it is used for the screening of clinical paranoia. A direct comparison of the ideas of reference sum-scores between cultures, however, may lead to an over-estimation of these milder forms of paranoia in some (non-western) cultures.

## Introduction

Research on the aetiology and treatment of paranoia has grown exponentially over the last few decades, resulting in the necessity to develop reliable and valid self-report instruments to quantify current levels of, and longitudinal changes in, paranoia. Consequently, several self-report questionnaires of paranoid beliefs have been developed for use in clinical and nonclinical populations, including for example the Paranoia Scale (Fenigstein & Vanable, 1992)^,^ Peters' Delusions Inventory (Peters, Joseph, & Garety, 1999), the Personal Experiences of Paranoia Scale (Ellett, Lopes, & Chadwick, 2003), the Paranoia Checklist (Freeman et al., 2005), and the Green Paranoid Thoughts Scale (Green et al., 2008). These questionnaires have refined the assessment of paranoid beliefs and have provided a sound base for aetiological, epidemiological, and intervention research.

Among these questionnaires, the Revised Green et al., Paranoid Thoughts Scale (R-GPTS; Freeman et al., 2021) stands out for multiple reasons. First, building on prior theoretical (Freeman et al., 2005) and empirical evidence (Moritz, Van Quaquebeke, & Lincoln, 2012) showing that paranoid thoughts are multifaceted, it includes putatively more common social evaluative concerns and *ideas of reference* and more severe *persecutory thoughts* as separate subscales. Second, its validation is based on a particularly large sample (i.e. 8386 non-clinical individuals and 2165 patients with psychosis; Freeman et al., 2021). Third, a recent systematic review (Statham, Emerson, & Rowse, 2019) identified the Green et al., Paranoid Thoughts Scale as the most valid and accurate questionnaire of paranoid thoughts in general population and clinical samples due to the fact that it covers the full range of mild to severe paranoid beliefs, has the most clearly defined construct underlying its items, and shows the comparatively best psychometric properties. Fourth, its validation included determining latent construct ranges that can be meaningfully interpreted as classes ranging from ‘average’ to ‘very severe’ levels of ideas of reference/persecutory thoughts. Finally, some of these class cut-offs correspond to validated cutoffs for clinical levels of paranoia (persecutory thoughts sum-score of 11) and likely persecutory delusions (persecutory thoughts sum-score of 18; Freeman et al., 2021). Specifically, the cut-off between mildly elevated and moderate levels of paranoia (i.e. ‘clinical levels of paranoia’, sum-score⩾11) correspond to the optimal point identified from receiver-operator curves to differentiate between patients with clinical levels of persecutory delusions and non-clinical participants (sensitivity = 0.93, specificity = 0.85). The cut-off between moderate and severe levels of paranoia (i.e. ‘likely persecutory delusions’, sum-score⩾18) in turn corresponds to the cut-off optimized for minimal false positives (specificity = 0.93) while also maintaining sufficient levels of sensitivity (0.81) to detect patients with clinical persecutory delusions (for more details, see Freeman et al., 2021).

Despite being comparatively novel, the R-GPTS has already been used in numerous studies including interventions to reduce paranoia (Brown, Waite, Rovira, Nickless, & Freeman, 2020; Freeman et al., 2022), epidemiological studies (Rek et al., 2022), and experimental studies (see Ellett et al., 2023b for a review) to explore the causal mechanisms of paranoia (Barnby, Mehta, & Moutoussis, 2022) in various countries. Furthermore, the R-GPTS has also already been translated into various languages, including French (Latteur, Larøi, & Bortolon, 2022), Polish (Kowalski, Marchlewska, Molenda, Górska, & Gawęda, 2020), as well as German and Chinese (Kingston et al., 2023a). With this multi-cultural, multi-lingual implementation of the R-GPTS comes the implicit assumption that its psychometric properties (including its UK-based cutoffs; Freeman et al., 2021) can be readily used in different contexts. To date, however, there have been no formal tests of the measurement invariance (i.e. equivalence of the assessed construct) of the R-GPTS across cultures and translated versions. Thus, we have yet to determine whether paranoia as assessed and quantified by the R-GPTS has the same meaning to people from different countries or whether language differences or differences in people's reaction to the item content preclude a direct comparison of scores across cultural groups. Commonly, three core components of measurement invariance are needed for a comparison of means of the latent constructs measured by a questionnaire (Putnick & Bornstein, 2016): First, *configural invariance* needs to be tested. In case of the R-GPTS, configural invariance is the equivalence of the underlying model of paranoia consisting of the two factors ideas of reference and persecutory thoughts. If a similar pattern of items loading on the respective factors can be established, it is then tested whether the item loadings are equal across group, i.e., whether the difference between two response options for any item is indicative of the same difference in the latent construct of ideas of reference/persecutory thoughts across cultural groups (*metric invariance*). Finally, *scalar invariance* is tested. Scalar invariance means that not only the loadings of items, but also their intercept is equal across the groups, meaning that the mean differences in the latent constructs of paranoia capture all mean differences in the variance the items share with their factor. In other words, no item introduces a systematic over- or underestimation of the latent construct in any of the cultural groups. Only if such a level of measurement invariance can be established is it possible to directly compare means between cultural groups or to establish the validity of cut-offs across cultures.

To address this gap in the literature, in this study, we examine the measurement invariance of the R-GPTS. Using data from an existing multinational cross-sectional survey with data from the UK, USA, Australia, Germany, and Hong Kong, we tested the R-GPTS for (1) configural invariance regarding its two-factor structure, followed by (2) metric invariance and scalar invariance, which would be prerequisites for meaningfully comparing sum-scores across countries. We hypothesized that the R-GPTS would show configural, metric, and scalar invariance.

However, if no invariance for the full R-GPTS was found, we aimed to explore (3) whether invariance can be established for the ideas of reference or persecutory thoughts subscale alone, and (4) whether invariance can be found within different countries and/or language versions of the R-GPTS.

## Methods

### Design and procedure

This study uses data from a cross-sectional online survey. Data were collected between February and March 2021 across the UK, USA, Australia, Germany, and Hong Kong on the topic of vaccine hesitancy and pandemic-specific paranoia as well as general suspiciousness; Kingston et al., 2023a, 2023b; Lincoln et al., 2022; So et al., 2022). Participants completed a questionnaire battery that included the R-GPTS, pandemic specific paranoid beliefs (pandemic paranoia scale; Kingston et al., 2023a; Ellett et al., 2023a), indicators of mental health in general (e.g. depression, anxiety, stress, worrying) as well as various resilience and risk factors (e.g. trauma experiences, core beliefs about one-self and others). In the current study, only the R-GPTS was analyzed.

Ethical approval was obtained separately from local ethics committees at each of the host sites. Potential participants were contacted by Qualtrics to take part. Consenting participants completed the questionnaires online via Qualtrics and were reimbursed via Qualtrics sampling services. Participants were required to respond to all questions on each page before progressing through the survey. Data accuracy was optimized by (1) including five attention check questions across the survey. Participants had to correctly respond to all five attention checks to be included. Moreover, (2) participants completing the survey at less than half of the median completion time and (3) participants with a geographical location outside the corresponding site location were excluded. Participants who did not fulfill the data accuracy requirements, did not give their informed consent to their data being used or dropped out without completing the full questionnaire battery were excluded at source by Qualtrics (excluded participants: *n* = 3555).

### Participants

Participants were recruited via Qualtrics using stratified quota sampling. Each sample was stratified to be representative of the respective general population in terms of sex, age, and level of education. Sample size was determined based on the minimum sample size to validate the newly constructed pandemic paranoia scale (Kingston et al., 2023a). A total of 2510 participants (UK *n* = 512, USA *n* = 535, Australia *n* = 502, Germany *n* = 516, and Hong Kong *n* = 445) met quota and quality assurance conditions and were included in the final sample. Sample characteristics are summarized in Table 1.

**Table 1.** Demographic and clinical sample characteristics by site

	UK (*n* = 512)	USA (*n* = 535)	Australia (*n* = 502)	Germany (*n* = 516)	Hong Kong (*n* = 445)
Age in years: M (s.d.)	41.91 (14.87)	47.65 (17.05)	44.75 (17.55)	42.00 (13.79)	39.64 (13.57)
Gender (%)					
Male	47.1%	46.4%	48.2%	49.2%	43.1%
Female	52.7%	52.7%	50.8%	50.0%	56.6%
Other/Genderqueer, TransMale/-Female	0%	1.0%	0.8%	0.8%	0.2%
Education					
Primary	0.4%	5.2%	0.8%	0.4%	2.5%
Secondary/equivalent	19.7%	0%	15.5%	59.7%	28.8%
A-level/equivalent	38.3%	34.4%	49.2%	12.8%	18.2%
University degree (Bachelor/Master/PhD)	41.7%	60.3%	34.5%	27.1%	50.6%
Income					
Under £ 18 500	15.6%	26.7%	22.9%	20.9%	8.5%
£ 18 500-£ 36 999	39.8%	25.0%	27.1%	28.3%	22.2%
£ 37 000-£ 55 999	23.6%	16.1%	13.3%	23.4%	28.8%
£ 56 000-£ 74 999	11.5%	10.1%	13.3%	14.7%	11.7%
£ 75 000-£ 92 999	4.7%	6.9%	12.4%	6.2%	13.9%
£ 93 000-£ 111 999	2.1%	7.5%	7.4%	3.3%	8.3%
£ 112 000 +	2.5%	7.7%	3.6%	3.1%	6.5%
Employment (%)					
Full/Part time employed	71.1%	49.7%	54.7%	68.0%	84.1%
Retired	10.4%	0%	16.9%	8.7%	3.6%
Unemployed/not working	6.9%	27.0%	10.2%	7.9%	2.3%
Home keeper/carer	5.7%	9.2%	7.2%	4.5%	1.3%
Disabled	1.6%	4.7%	6.0%	2.5%	0%
Training/school	4.3%	8.2%	4.0%	8.3%	8.8%
Current psychiatric diagnosis (% yes)	12.3%	22.4%	41.8%	20.0%	7.2%
R-GPTS: M (s.d.)					
Ideas of reference	6.46 (8.59)	6.77 (8.69)	11.73 (8.52)	7.13 (8.60)	9.17 (7.07)
Persecutory thoughts	6.03 (9.66)	6.85 (10.42)	12.01 (11.02)	5.72 (9.11)	8.52 (9.67)

### Measure

#### R-GPTS

The Revised Green et al. Paranoid Thoughts Scale (Freeman et al., 2021) is an 18-item measure that comprises two subscales: ideas of reference (8 items, e.g. ‘I have been upset by friends and colleagues judging me critically’) and paranoia/persecutory thoughts (10 items, .e.g. ‘I was sure someone wanted to hurt me’). Items are rated on a 5-point scale of 0 – not at all to 4 – totally. Sum-scores for ideas of reference (range: 0–32) and persecutory thoughts (range: 0–40) are usually calculated and provide the base for categorization from ‘average’ to ‘very severe’. For the persecutory thoughts subscale, a sum-score of 18 or more is indicative of persecutory delusions. In the current sample, Cronbach's alpha was excellent for both subscales (ideas of reference: *α* = 0.94; persecutory thoughts: *α* = 0.96).

For the UK, USA, and Australia, we used the original English version of the R-GPTS. For the German site, we used an existing translation of the GPTS as a starting point (Watzke & Schwenke, 2014) for the translation of the R-GPTS. Translated and original items were compared for any discrepancies and changes to the wording of the German version were added based on consensus between TML and a graduate level psychologist from the German site. Reliability of the German R-GPTS has been tested in two concurrent, independent studies that are as yet unpublished (see Schönig, Krkovic, & Lincoln, 2022a, 2022b for corresponding pre-registrations). Preliminary results from these studies (sample sizes *n* = 50, and *n* = 31, respectively) showed good reliability for both ideas of reference (0.83 ⩽ *α* ⩽ 0.89), persecutory thoughts (0.87 ⩽ *α* = 0.91), and the full scale (0.93 ⩽ *α* ⩽ 0.93) as well as high correlations with the PSYRATS delusion subscale (*n* = 31, 0.55 ⩽ *r* ⩽ 0.61). For the Hong Kong site, we translated the R-GPTS to Traditional Chinese (i.e. the writing system that is used in both HK and Taiwan): First, the English scale was translated into Chinese, followed by a back-translation of the Chinese version into English, and a check for consistency between the back-translated version and the original English version. After a discussion of discrepancies, fine-tuning of the wording for the translated version resulted in the final Chinese version of the R-GPTS. The translation and back-translation were conducted independently by bilingual, graduate-level psychologists. Comparison of the original *v.* back-translated version and decisions for final changes were based on consensus between the two translating psychologists, with SHS acting as consultant if needed. The traditional Chinese version of the R-GPTS has been used in another study (Chau et al., 2022), where it showed an excellent Cronbach's alpha (*α* = 0.95). The German and Traditional Chinese versions of the R-GPTS have been added as an online Supplement to this article.

### Strategy for data analyses

Analyses were conducted using R 4.2.2 and the R-package lavaan (Rosseel, 2012). For all analyses, we calculated confirmatory factor analyses with Satorra-Bentler scaled test statistic. We tested measurement invariance in the following three steps: (1) configural invariance, (2a) invariance of loading (i.e. metric invariance), and (2b) invariance of loadings and intercepts (i.e. scalar invariance). Configural invariance was determined by the three indicators CFI (good fit CFI > 0.95, sufficient fit CFI > 0.90), RMSEA (good fit RMSEA < 0.06, sufficient fit RMSEA < 0.08), and SRMR (SRMR < 0.08; for information of thresholds on all indicators, see Chen, 2007). Metric invariance was assessed on the basis of differences in fit indices in comparison to the configural invariance model, using the cutoffs *ΔCFI* > −0.010, *ΔRMSEA* < 0.015, and *Δ*SRMR < 0.030 as an indication of metric invariance (Meade, Johnson, & Braddy, 2008). For the assessment of scalar invariance, differences in fit indices in comparison to the metric invariance model were used, with the cut-offs *ΔCFI* > −0.010, *ΔRMSEA* < 0.015, and *Δ*SRMR < 0.015 indicating scalar invariance (Meade et al., 2008).

First, we tested invariance for the full 18-item R-GPTS with the full sample. Second, if indicators of non-invariance were found, we followed up with secondary analyses of invariance of the R-GPTS ideas of reference and R-GPTS persecutory thoughts subscales to determine the source of invariance in relation to specific subscales.

Finally, (3) we repeated the invariance analyses in language and culture specific subsamples. We performed a measurement invariance analyses across the English speaking countries (UK, USA, Australia) to explore whether the R-GPTS shows invariance across countries with the same (English) version of the scale. Next, we followed up by adding either the German sample or the Hong Kong sample in two additional invariance analyses to explore if and how invariance differs across different language/culture-dyads.

## Results

### Full sample invariance analyses

Results of the invariance analyses of the full sample (*N* = 2510) are summarized in Table 2. Measurement invariance analyses of the two-factor model (1. ideas of reference, 2. persecutory thoughts) yielded sufficient fit for the configural and metric invariance model. For scalar invariance, invariance was supported by RMSEA (RMSEA = 0.62, *ΔRMSEA* = 0.006) and SRMR (*SRMR* = 0.066, *ΔSRMR* = 0.005), but not by CFI (*CFI* = 0.930, *ΔCFI* = 0.018).

**Table 2.** Measurement invariance confirmatory factor analyses on the full five-site sample (*N* = 2510)

				Threshold ΔCFI > −0.010		Threshold ΔRMSEA < 0.015		Thresholds ΔSRMR < 0.030 metric ΔSRMR < 0.015 scalar	
Model	χ^2^	χ^2^Scaled	df	CFI	ΔCFI	RMSEA	ΔRMSEA	SRMR	ΔSRMR
Full R-GPTS two factor model									
Configural invariance	3909.35	1723.69	670	0.953		0.056		0.039	
Metric invariance	4152.61	1892.93	734	0.948	−0.005	0.056	0.000	0.061	0.022
Scalar invariance	4828.88	2357.36	798	**0.930**	**−0.018**	0.062	0.006	0.066	0.005
Ideas of reference									
Configural invariance	639.15	312.93	100	0.977		0.065		0.029	
Metric invariance	730.26	395.94	128	0.971	−0.006	0.065	0.000	0.056	0.027
Scalar invariance	1217.57	747.95	156	**0.937**	**−0.034**	**0.087**	**0.022**	**0.073**	**0.017**
Persecutory thoughts									
Configural invariance	1381.28	482.62	35	0.970		0.059		0.029	
Metric invariance	1539.54	579.04	211	0.964	−0.006	0.059	0.000	0.058	0.029
Scalar invariance	1729.09	724.77	247	**0.953**	**−0.011**	0.062	0.003	0.061	0.003

*Note*. Cells printed in bold denote indicators of non-invariance.

When analyzing the subscales of the R-GPTS separately, we again found sufficient fit for configural and metric invariance for both subscales (see Table 2). There was insufficient fit for scalar invariance, however, with one indicator showing insufficient fit for the persecutory thoughts subscale (*CFI* = 0.953, *ΔCFI* = 0.011) and all three indicators showing insufficient fit for the ideas of reference subscale (*CFI* = 0.937, *ΔCFI* = 0.034; RMSEA = 0.087, *ΔRMSEA* = 0.022; *SRMR* = 0.073, *ΔSRMR* = 0.017).

### Language subsample invariance analyses

When analyzing the subsamples from the English speaking countries (UK, USA, and Australia), invariance analysis yielded sufficient fit for configural, metric, and scalar invariance (see Table 3). These results were similar, when the two subscales (ideas of reference and persecutory thoughts) were analyzed separately (see online Supplement).

**Table 3.** Measurement invariance results for the full R-GPTS two-factor model in language and culture subsamples

				Threshold ΔCFI > −0.010		Threshold ΔRMSEA < 0.015		Thresholds ΔSRMR < 0.030 metric ΔSRMR < 0.015 scalar	
Model	χ^2^	χ^2^Scaled	df	CFI	ΔCFI	RMSEA	ΔRMSEA	SRMR	ΔSRMR
English samples (UK, USA, AUS) – *N* = 1549									
Configural invariance	2297.20	977.54	402	0.959		0.053		0.036	
Metric invariance	2407.65	1059.79	434	0.955	−0.004	0.053	0.000	0.054	0.018
Scalar invariance	2500.93	1150.45	466	0.951	−0.004	0.053	0.000	0.055	0.001
English/German samples – *N* = 2065									
Configural invariance	3088.76	1300.25	536	0.956		0.053	0.053	0.038	
Metric invariance	3266.23	1421.07	584	0.952	−0.004	0.053	0.000	0.058	0.020
Scalar invariance	3499.25	1606.90	632	0.944	−0.008	0.055	0.002	0.060	0.002
English/Hong Kong samples – *N* = 1994									
Configural invariance	3117.78	1403.16	536	0.954		0.057		0.039	
Metric invariance	3295.80	1534.59	584	0.950	−0.004	0.057	0.000	0.058	0.019
Scalar invariance	3758.47	1862.41	632	0.935	**−0.015**	0.062	0.005	0.064	0.006

*Note*. Cells printed in bold denote indicators of non-invariance.

When analyzing the English and German subsamples without the Hong Kong sample, the full scale analyses yielded configural, metric, or scalar invariance (see Table 3). When analyzing the subscales separately, we found invariance for the persecutory thoughts subscale, whereas scalar invariance was not found for the ideas of reference subscale (χ^2^(122) = 658.83, χ^2^(122)_scaled_ = 375.12, CFI = 0.967, ΔCFI = 0.013, RMSEA = 0.063, ΔRMSEA = 0.008, SRMR = 0.056, ΔSRMR = 0.006, see online Supplement for more details).

Analyses of the English and Hong Kong subsamples yielded configural and metric invariance, but difference in CFI did not support scalar invariance for the full scale (*CFI* = 0.909, *ΔCFI* = 0.026). The separate analyses of the subscales yielded non-invariance for the ideas of reference subscale (χ^2^(122) = 944.96, χ^2^(122)_scaled_ = 584.61, CFI = 0.939, ΔCFI = 0.029, RMSEA = 0.087, ΔRMSEA = 0.018, SRMR = 0.074, ΔSRMR = 0.015). For the persecutory thoughts subscale, all indicators support scalar invariance (χ^2^(194) = 1282.21, χ^2^(194)_scaled_ = 554.27, CFI = 0.960, ΔCFI = 0.008, RMSEA = 0.061, ΔRMSEA = 0.003, SRMR = 0.057, ΔSRMR = 0.002, see online Supplement).

To better understand the nature of the invariance, we repeated the factor analyses while freeing one item intercept, respectively, and comparing the pooled estimate for the item intercept with the freely estimated intercept for the Hong Kong sample. The resulting comparisons yielded substantially higher intercept estimates in the Hong Kong *v.* pooled sample the items 7 (‘I believed that certain people were not what they seemed’, Δintercept = 0.57) and 3 (‘I have been upset by friends and colleagues judging me critically’, Δintercept = 0.31) and a lower estimate for item 5 (‘I have been thinking a lot about people avoiding me’, Δintercept = −0.28).

## Discussion

In this study, we explored the measurement invariance of the Revised Green at al. Paranoid Thoughts Scale (R-GPTS). We found configural invariance, supporting the notion that paranoia can be differentiated in milder, more common symptoms that encompass ideas of reference and other forms of social evaluative concerns on the one hand, and more severe persecutory thoughts on the other hand (Freeman et al., 2021). These findings are consistent with continuum models (Strauss, 1969; Verdoux & van Os, 2002) and provide further support for a multifaceted model of paranoid beliefs (Green et al., 2008; Moritz et al., 2012). We add to this by showing empirically that a two factor model of paranoid beliefs comprising social evaluative beliefs and ideas of reference on the one hand and persecutory beliefs on the other hand appears to be applicable across cultures.

Regarding the more severe form of paranoid beliefs, persecutory thoughts, we further found substantial evidence for scalar invariance for the corresponding subscale of the R-GPTS. This is no small feat, since prior tests of paranoia measures across cultures showed no scalar invariance for paranoia (sub)scales (Heuvelman, Nazroo, & Rai, 2018; Jaya et al., 2022), which Jaya et al. (2022) highlight as an indication of the ‘general difficulty of constructing an invariant measure of paranoia’. Scalar invariance for the persecutory thoughts subscale in particular is of substantial practical importance, as persecutory thoughts scores have previously been established as the basis for cut-offs in screening for clinical levels of paranoid delusions (Freeman et al., 2021). With evidence for scalar invariance for this subscale, future studies can now test for differences in mean latent construct values across languages/cultures in order to formally verify the validity of the existing cut-off (sum score ⩾ 18) cross-culturally and in other populations than the UK. Assuming that the same level of (the latent of construct of) persecutory beliefs is indicative of the same risk for a psychotic disorder across cultures, researchers and practitioners may provisionally work with the established UK cut-off. However, it needs noting that this equivalence has not been directly tested. Rather, our initial results regarding the invariance of the R-GPTS persecutory thoughts subscale are a promising first step toward cross-culturally valid cut-offs for this self-report questionnaire. What is needed to complete validation of the cut-offs is a comparison of mean latent scores between samples that are optimized for the testing of cut-offs (i.e. samples stratified for mental health status).

Regarding the less severe form of paranoid beliefs, ideas of reference, we found metric but not scalar invariance. In other words, agreeing more with the R-GPTS ideas of reference items corresponds with equal gradual increases of the latent construct of ideas of reference across cultures. However, the intercept of these items varies across cultures, meaning that individual items can add an over- or underestimation of the latent construct of ideas of reference. In practice, this means that comparison of the latent mean scores of the R-GPTS ideas of reference factor between samples from different cultures is at risk for biased results. Interestingly, this scalar invariance was limited to the comparison of the three English-speaking samples (UK, USA, and Australia) and the Hong Kong sample. When only the English-speaking or the English-speaking and German-speaking samples were used for invariance analyses, we found no indication of non-invariance for ideas of reference. Possibly, cultural differences in trust-formation in Western *v.* Eastern societies (Yuki, Maddux, Brewer, & Takemura, 2005) that may be correlated with the degree of collectivism of the respective culture (Westjohn, Magnusson, Franke, & Peng, 2022) influence what constitutes the norm/absence of elevated social-evaluative concerns, though this would need to be established in future research. In line with this, Jaya et al. (2022) already pointed out that the assessment of paranoia in self-report is intertwined with objective levels of (social) threat. These levels may greatly differ as a function of geographical location, culture-specific social norms, and/or differences in the social status of certain minorities that consequently face higher or lower levels of threat in different places. The combination of non-invariance for ideas of reference and scalar invariance for persecutory thoughts could point toward qualitative differences in the building blocks of the hierarchy of paranoia. In particular, what constitutes an elevated level of more mundane forms of mistrust may vary with cultural standards, whereas the starting point for what can be considered more severe or pathological forms of paranoia is universal and appears to be largely unaffected by cultural differences. In the case of ideas of reference, we found overall more items with an increased than a lower intercept when comparing with the Hong Kong sample. Specifically, participants from Hong Kong show higher scores in items that deal with a not directly disclosed social judgment by others (i.e. ‘believing others are not what they seem’ and ‘being upset by being critically judged’). This sample-specific tendency is unrelated to the ideas of reference. Instead, it may be a result of a culture-specific difference in norms and expectation in social interactions. Thus, a person from Hong Kong is likely to score higher in ideas of reference than a person from the UK, USA, or Australia, even when both participants have the same true value. At the same time, a minority of items with a lower intercept in Hong Kong *v.* the other samples (for the item dealing with ‘concerns regarding other people avoiding the respondent’) make it difficult to predict the exact direction of the biased estimate for an individual participant.

### Strengths and limitations

While the recruitment with stratified quota sampling constitutes a major strength of this study in terms of generalizability, it needs noting that the sampling was conducted via recruitment in the Panel samples provided by Qualtrics, impacting the representativeness of the sample to some degree. We purposefully sampled region-specific representative samples, yet there are groups that could nonetheless be under-represented, such as those with limited access to a computer or the internet. It needs noting though that a study comparing different online-recruitment procedures found Qualtrics to be the best option in terms of achieving approximate demographic representativeness and geographic representativeness in a high-income country (when compared to equally common commercial providers such as MTurk or Facebook-advertisement; Boas, Christenson, & Glick, 2020). Divergences from representativeness are gradual, and indications of some limitations regarding the participant pool have been documented for people with high income and people of higher age (i.e. aged 50 and older, Miller, Guidry, Dahman, & Thomson, 2020). To our knowledge, however, there is no existing research detailing potential selection bias in terms of clinical variables. Related to this, it needs noting that invariance-assessments of non-clinical and clinical samples (i.e. patients with psychosis) might show diverging results, necessitating future tests in clinical samples to further verify our initial findings. Furthermore, the lack of a more objective criterion to validate the R-GPTS, in particular other self-report- or interview-based assessment of paranoid delusions, prevented us from further criterion validation with the present data. Further, our data may have been impacted by the Covid-19 pandemic, given the timing of data collection. With COVID-19 affecting the five international sites differently at the time of recruitment, it is possible that the mean scores of persecutory thoughts may have temporarily shifted to different degrees in the sample, preventing us from disentangling permanent mean differences in persecutory thoughts across the five sites from transitory fluctuations due to the Covid-19 pandemic. Ideally, future research should collect data at several assessment points in different cultures to disentangle fluctuations in latent means due to, for example public or political crises, from stable intercultural differences in mean persecutory thoughts. Such an effort would allow for either the generalization of the established UK cut-offs or the generation of improved cross-cultural or culture-specific cut-offs. Finally, while there was some cultural variation in the samples analyzed, all five sites constitute high-income regions, so a more extensive follow-up to this study is needed to further verify invariance across high, middle, and low income countries.

## Conclusion

This analysis of measurement invariance of the R-GPTS showed that its persecutory thoughts subscale is a reliable and valid self-report measure of severe levels of paranoia, which provides the first step toward an unbiased assessment across cultures and cross-cultural verification of cut-off criteria. At the same time, our analyses revealed that a direct comparison of the ideas of reference subscale sum-scores between cultures may lead to an over-estimation of these milder forms of paranoia in some (non-western) cultures. In sum, the R-GPTS constitutes a valuable tool for researchers and practitioners assessing, treating, and exploring the phenomenon of paranoid beliefs.

## Supporting information

Schlier et al. supplementary material Schlier et al. supplementary material

## Supplementary material

The supplementary material for this article can be found at https://doi.org/10.1017/S0033291724000072

## References

[ref1] Barnby, J. M., Mehta, M. A., & Moutoussis, M. (2022). The computational relationship between reinforcement learning, social inference, and paranoia. PLOS Computational Biology, 18(7), e1010326. 10.1371/journal.pcbi.1010326 35877675 PMC9352206

[ref2] Boas, T. C., Christenson, D. P., & Glick, D. M. (2020). Recruiting large online samples in the United States and India: Facebook, mechanical turk, and qualtrics. Political Science Research and Methods, 8(2), 232–250. 10.1017/psrm.2018.28

[ref3] Brown, P., Waite, F., Rovira, A., Nickless, A., & Freeman, D. (2020). Virtual reality clinical-experimental tests of compassion treatment techniques to reduce paranoia. Scientific Reports, 10(1), 8547. 10.1038/s41598-020-64957-7 32444619 PMC7244556

[ref4] Chau, A. K. C., So, S. H. W., Sun, X., Zhu, C., Chiu, C. D., Chan, R. C., & Leung, P. W. (2022). The co-occurrence of multidimensional loneliness with depression, social anxiety and paranoia in non-clinical young adults: A latent profile analysis. Frontiers in Psychiatry, 13, 931558. 10.3389/fpsyt.2022.931558 36186883 PMC9517946

[ref5] Chen, F. F. (2007). Sensitivity of goodness of fit indexes to lack of measurement invariance. Structural Equation Modeling: A Multidisciplinary Journal, 14(3), 464–504. 10.1080/10705510701301834

[ref6] Ellett, L., Lopes, B., & Chadwick, P. (2003). Paranoia in a nonclinical population of college students. The Journal of Nervous and Mental Disease, 191(7), 425–430. 10.1097/01.NMD.0000081646.33030.EF 12891088

[ref7] Ellett, L., Schlier, B., Kingston, J. L., Zhu, C., So, S. H., Lincoln, T. M., … Gaudiano, B. A. (2023a). Pandemic paranoia in the general population: International prevalence and sociodemographic profile. Psychological Medicine, 53(12), 5748–5755. 10.1017/S0033291722002975. 36065655 PMC9530376

[ref8] Ellett, L., Varese, F., Owens, J., Rafiq, S., Penn, G., & Berry, K. (2023b). Experimental studies of paranoid thinking in clinical and nonclinical populations: A systematic review and meta-analysis. Psychological Medicine, 52, 5933–5944. 10.1017/S0033291723001708 PMC10520588 37427557

[ref9] Fenigstein, A., & Vanable, P. A. (1992). Paranoia and self-consciousness. Journal of Personality and Social Psychology, 62(1), 129–138. 10.1037/0022-3514.62.1.129 1538311

[ref10] Freeman, D., Garety, P. A., Bebbington, P. E., Smith, B., Rollinson, R., Kuipers, E., … Katarzyna, R. (2005). Psychological investigation of the structure of paranoia in a non-clinical population psychological investigation of the structure of paranoia in a non-clinical population. The British Journal of Psychiatry, 186(5), 427–435. 10.1192/bjp.186.5.427 15863749

[ref11] Freeman, D., Lambe, S., Kabir, T., Petit, A., Rosebrock, L., Yu, L.-M., … West, J. (2022). Automated virtual reality therapy to treat agoraphobic avoidance and distress in patients with psychosis (gameChange): A multicentre, parallel-group, single-blind, randomised, controlled trial in England with mediation and moderation analyses. The Lancet Psychiatry, 9(5), 375–388. 10.1016/S2215-0366(22)00060-8 35395204 PMC9010306

[ref12] Freeman, D., Loe, B. S., Kingdon, D., Startup, H., Molodynski, A., Rosebrock, L., … Bird, J. C. (2021). The revised Green *et al*., Paranoid Thoughts Scale (R-GPTS): Psychometric properties, severity ranges, and clinical cut-offs. Psychological Medicine, 51(2), 244–253. 10.1017/S0033291719003155 31744588 PMC7893506

[ref13] Green, C. E. L., Freeman, D., Kuipers, E., Bebbington, P., Fowler, D., Dunn, G., Garety, P. A. (2008). Measuring ideas of persecution and social reference: The Green *et al*. paranoid thought scales (GPTS). Psychological Medicine, 38(1), 101–111. 10.1017/S0033291707001638 17903336

[ref14] Heuvelman, H., Nazroo, J., & Rai, D. (2018). Investigating ethnic variations in reporting of psychotic symptoms: A multiple-group confirmatory factor analysis of the psychosis screening questionnaire. Psychological Medicine, 48(16), 2757–2765. 10.1017/s0033291718000399 29526172

[ref15] Jaya, E. S., Wüsten, C., Alizadeh, B. Z., Van Amelsvoort, T., Bartels-Velthuis, A. A., Van Beveren, N. J.…Lincoln, T. M. (2022). Comparing psychotic experiences in low-and-middle-income-countries and high-income-countries with a focus on measurement invariance. Psychological Medicine, 52(8), 1509–1516. 10.1017/S0033291720003323. 33023691

[ref16] Kingston, J. L., Schlier, B., Ellett, L., So, S. H., Gaudiano, B. A., Morris, E. M. J., & Lincoln, T. M. (2023a). The pandemic paranoia scale (PPS): Factor structure and measurement invariance across languages. Psychological Medicine, 53(6), 2652–2661. 10.1017/S0033291721004633 34879896 PMC8712962

[ref17] Kingston, J. L., Schlier, B., Lincoln, T., So, S. H., Gaudiano, B. A., Morris, E. M. J., … Ellett, L. (2023b). Paranoid thinking as a function of minority group Status and intersectionality: An international examination of the role of negative beliefs. Schizophrenia Bulletin, 49(4), 1078–1087. 10.1093/schbul/sbad027 36940411 PMC10318883

[ref18] Kowalski, J., Marchlewska, M., Molenda, Z., Górska, P., & Gawęda, Ł (2020). Adherence to safety and self-isolation guidelines, conspiracy and paranoia-like beliefs during COVID-19 pandemic in Poland - associations and moderators. Psychiatry Research, 294, 113540. 10.1016/j.psychres.2020.113540 33142144 PMC7590639

[ref19] Latteur, A., Larøi, F., Bortolon, C. (2022). Translation and validation of the French version of the revised Green *et al*., paranoid thoughts scale (R-GPTS) in two samples: Non-clinical and clinical adults. Psychologica Belgica, 62(1), 208–217. 10.5334/pb.1134 35692717 PMC9165671

[ref20] Lincoln, T. M., Schlier, B., Strakeljahn, F., Gaudiano, B. A., So, S. H., Kingston, J., … Ellett, L. (2022). Taking a machine learning approach to optimize prediction of vaccine hesitancy in high income countries. Scientific Reports, 12(1), 2055. 10.1038/s41598-022-05915-3 35136120 PMC8827083

[ref21] Meade, A. W., Johnson, E. C., & Braddy, P. W. (2008). Power and sensitivity of alternative fit indices in tests of measurement invariance. Journal of Applied Psychology, 93(3), 568–592. 10.1037/0021-9010.93.3.568 18457487

[ref22] Miller, C. A., Guidry, J. P., Dahman, B., & Thomson, M. D. (2020). A tale of two diverse qualtrics samples: Information for online survey researchers. Cancer Epidemiology, Biomarkers & Prevention, 29(4), 731–735. 10.1158/1055-9965.EPI-19-0846 PMC8758054 32066616

[ref23] Moritz, S., Van Quaquebeke, N., & Lincoln, T. M. (2012). Jumping to conclusions is associated with paranoia but not general suspiciousness: A comparison of two versions of the probabilistic reasoning paradigm. Schizophrenia Research and Treatment, 2012, 1–9. 10.1155/2012/384039 PMC3483676 23125930

[ref24] Peters, E. R., Joseph, S. A., Garety, P. A. (1999). Measurement of delusional ideation in the normal population: Introducing the PDI (Peters *et al*. delusions inventory). Schizophrenia Bulletin, 25(3), 553–576. 10.1093/oxfordjournals.schbul.a033401 10478789

[ref25] Putnick, D. L., & Bornstein, M. H. (2016). Measurement invariance conventions and reporting: The state of the art and future directions for psychological research. Developmental Review, 41, 71–90. 10.1016/j.dr.2016.06.004 27942093 PMC5145197

[ref26] Rek, S. V., Freeman, D., Reinhard, M. A., Bühner, M., Grosen, S., Falkai, P., … Padberg, F. (2022). Differential psychological response to the COVID-19 pandemic in psychiatric inpatients compared to a non-clinical population from Germany. European Archives of Psychiatry and Clinical Neuroscience, 272(1), 67–79. 10.1007/s00406-021-01291-7 34268618 PMC8282176

[ref27] Rosseel, Y. (2012). lavaan: An R package for structural equation modeling. Journal of Statistical Software, 48(2), 1–36.

[ref28] Schönig, S., Krkovic, K., & Lincoln, T. M. (2022a). Safety behaviours in paranoid individuals' daily lives. 10.17605/OSF.IO/8TCHN

[ref29] Schönig, S., Krkovic, K., & Lincoln, T. M. (2022b). The role of safety behaviours for the maintenance of paranoid beliefs. 10.17605/OSF.IO/ZGX92

[ref30] So, S. H., Zhu, C., Lincoln, T. M., Gaudiano, B. A., Kingston, J. L., Ellett, L., & Morris, E. M. J. (2022). Pandemic paranoia, general paranoia, and their relationships with worry and beliefs about self/others – A multi-site latent class analysis. Schizophrenia Research, 241, 122–129. 10.1016/j.schres.2022.01.045 35121437

[ref31] Statham, V., Emerson, L.-M., & Rowse, G. (2019). A systematic review of self-report measures of paranoia. Psychological Assessment, 31(2), 139–158. 10.1037/pas0000645 30234319

[ref32] Strauss, J. S. (1969). Hallucinations and delusions as points on continua function. Archives of General Psychiatry, 21(5), 581. 10.1001/archpsyc.1969.01740230069010 5823480

[ref33] Verdoux, H., & van Os, J. (2002). Psychotic symptoms in non-clinical populations and the continuum of psychosis. Schizophrenia Research, 54(1–2), 59–65. 10.1016/S0920-9964(01)00352-8 11853979

[ref34] Watzke, S., & Schwenke, J. (2014). Irrationale Überzeugungen und paranoia. Nervenheilkunde, 33(09), 617–625. 10.1055/s-0038-1627720

[ref35] Westjohn, S. A., Magnusson, P., Franke, G. R., & Peng, Y. (2022). Trust propensity across cultures: The role of collectivism. Journal of International Marketing, 30(1), 1–17. 10.1177/1069031X211036688

[ref36] Yuki, M., Maddux, W. W., Brewer, M. B., & Takemura, K. (2005). Cross-cultural differences in relationship- and group-based trust. Personality and Social Psychology Bulletin, 31(1), 48–62. 10.1177/0146167204271305 15574661

