# Supplementary material for: Cross-cultural validation of the revised Green et al., paranoid thoughts scale
Source: Psychol Med. 2024 Feb 5;54(9):1985–91. doi: 10.1017/S0033291724000072 (PMC11413342; doi:10.1017/S0033291724000072)
Supplement: Schlier et al. supplementary material [file S0033291724000072sup001.docx]

Online Supplements

Table S1

Measurement invariance confirmatory factor analyses on the three site sample (UK, USA, Australia)

|  |  | | | Threshold  Δ >0.01 | | Threshold  Δ>0.015 | | Thresholds  Δ>0.03 metric  Δ>0.01 scalar | |
| --- | --- | --- | --- | --- | --- | --- | --- | --- | --- |
| Model | χ² | χ²Scaled | df | CFI | ΔCFI | RMSEA | ΔRMSEA | SRMR | ΔSRMR |
| Full RGPTS two factor model | |  |  |  |  |  |  |  |  |
| Configural invariance | 2297.20 | 977.54 | 402 | 0.959 |  | 0.053 |  | 0.036 |  |
| Metric invariance | 2407.65 | 1059.79 | 434 | 0.955 | 0.004 | 0.053 | 0.000 | 0.054 | 0.018 |
| Scalar invariance | 2500.93 | 1150.45 | 466 | 0.951 | 0.004 | 0.053 | 0.000 | 0.055 | 0.001 |
| Ideas of reference |  |  |  |  |  |  |  |  |  |
| Configural invariance | 337.28 | 158.37 | 60 | 0.983 |  | 0.056 |  | 0.025 |  |
| Metric invariance | 385.09 | 198.93 | 74 | 0.979 | 0.004 | 0.057 | 0.001 | 0.050 | 0.025 |
| Scalar invariance | 440.01 | 247.46 | 88 | 0.973 | 0.006 | 0.059 | 0.002 | 0.053 | 0.003 |
| Persecutory beliefs |  |  |  |  |  |  |  |  |  |
| Configural invariance | 777.02 | 268.06 | 105 | 0.975 |  | 0.055 |  | 0.027 |  |
| Metric invariance | 838.79 | 312.27 | 123 | 0.971 | 0.004 | 0.055 | 0.000 | 0.048 | 0.021 |
| Scalar invariance | 879.41 | 356.84 | 141 | 0.967 | 0.004 | 0.054 | -0.001 | 0.049 | 0.001 |

Note. Cells printed in bold denote indicators of non-invariance

Table S2

Measurement invariance confirmatory factor analyses on the English and German samples (UK, USA, Australia, Germany)

|  |  | | | Threshold  Δ >0.01 | | Threshold  Δ>0.015 | | Thresholds  Δ>0.03 metric  Δ>0.01 scalar | |
| --- | --- | --- | --- | --- | --- | --- | --- | --- | --- |
| Model | χ² | χ²Scaled | df | CFI | ΔCFI | RMSEA | ΔRMSEA | SRMR | ΔSRMR |
| Full RGPTS two factor model | |  |  |  |  |  |  |  |  |
| Configural invariance | 3088.76 | 1300.25 | 536 | 0.956 |  | 0.053 |  | 0.038 |  |
| Metric invariance | 3266.23 | 1421.07 | 584 | 0.952 | 0.004 | 0.053 | 0.000 | 0.058 | 0.020 |
| Scalar invariance | 3499.25 | 1606.90 | 632 | 0.944 | 0.008 | 0.055 | 0.002 | 0.060 | 0.002 |
| Ideas of reference |  |  |  |  |  |  |  |  |  |
| Configural invariance | 433.14 | 200.69 | 80 | 0.984 |  | 0.054 |  | 0.024 |  |
| Metric invariance | 501.75 | 257.68 | 101 | 0.980 | 0.004 | 0.055 | -0.001 | 0.050 | -0.026 |
| Scalar invariance | 658.83 | 375.12 | 122 | 0.967 | **0.013** | 0.063 | 0.008 | 0.056 | 0.006 |
| Persecutory beliefs |  |  |  |  |  |  |  |  |  |
| Configural invariance | 1124.57 | 376.24 | 140 | 0.969 |  | 0.057 |  | 0.029 |  |
| Metric invariance | 1229.32 | 441.94 | 167 | 0.964 | 0.005 | 0.056 | -0.001 | 0.055 | 0.026 |
| Scalar invariance | 1308.23 | 519.07 | 194 | 0.958 | 0.006 | 0.057 | 0.001 | 0.056 | 0.001 |

Note. Cells printed in bold denote indicators of non-invariance

Table S3

Measurement invariance confirmatory factor analyses on the English and Hong Kong samples (UK, USA, Australia, HK)

|  |  | | | Threshold  Δ >0.01 | | Threshold  Δ>0.015 | | Thresholds  Δ>0.03 metric  Δ>0.01 scalar | |
| --- | --- | --- | --- | --- | --- | --- | --- | --- | --- |
| Model | χ² | χ²Scaled | df | CFI | ΔCFI | RMSEA | ΔRMSEA | SRMR | ΔSRMR |
| Full RGPTS two factor model | |  |  |  |  |  |  |  |  |
| Configural invariance | 3117.78 | 1403.16 | 536 | 0.954 |  | 0.057 |  | 0.039 |  |
| Metric invariance | 3295.80 | 1534.59 | 584 | 0.950 | 0.004 | 0.057 | 0.000 | 0.058 | 0.019 |
| Scalar invariance | 3758.47 | 1862.41 | 632 | 0.935 | **0.015** | 0.062 | 0.005 | 0.064 | 0.006 |
| Ideas of reference |  |  |  |  |  |  |  |  |  |
| Configural invariance | 543.29 | 272.72 | 80 | 0.974 |  | 0.070 |  | 0.030 |  |
| Metric invariance | 615.90 | 340.46 | 101 | 0.968 | 0.006 | 0.069 | -0.001 | 0.059 | 0.029 |
| Scalar invariance | 944.96 | 584.61 | 122 | 0.939 | **0.029** | 0.087 | **0.018** | 0.074 | **0.015** |
| Partial Scalar invariance  (3 items^a^) | 647.39 | 393.79 | 113 | 0.963 | 0.005 | 0.071 | 0.002 | 0.061 | 0.002 |
| Partial Scalar invariance  (2 items^b^) | 748.49 | 445.83 | 116 | 0.956 | **0.012** | 0.076 | .007 | 0.063 | 0.004 |
| Persecutory beliefs |  |  |  |  |  |  |  |  |  |
| Configural invariance | 1033.725 | 374.19 | 140 | 0.974 |  | 0.058 |  | 0.027 |  |
| Metric invariance | 1148.459 | 449.188 | 167 | 0.968 | 0.006 | 0.058 | 0.000 | 0.055 | 0.028 |
| Scalar invariance | 1282.21 | 554.272 | 194 | 0.960 | 0.008 | 0.061 | 0.003 | 0.057 | 0.002 |

Note. Cells printed in bold denote indicators of non-invariance; a = partial invariance (3 items) was tested with items intercepts for items 3, 5, and 7 of the R-GPTS ideas of reference scale freed; b = partial invariance (3 items) was tested with items intercepts for items 3 and 7 of the R-GPTS ideas of reference scale freed.

**Traditional Chinese version of the R-GPTS**

**Revised Green et al. Paranoid Thoughts Scale (R-GPTS)**

**Revised妄想思維量表**

請仔細閱讀以下句子。它們關於你**過去兩個星期**對他人可能出現過的想法及感覺。 請回想你在**過去兩個星期**的經歷，並透過選出數字0-4中來表示該感受的程度。

(請不要根據你在藥物影響下的經歷而作出判斷)

|  | Not at all  完全沒有 |  |  |  | Totally  完全如此 |
| --- | --- | --- | --- | --- | --- |
| Part A |  |  |  |  |  |
| 1. 我花時間想著朋友們如何説我的閒話。 | 0 | 1 | 2 | 3 | 4 |
| 2. 我經常聽見人們談及我。 | 0 | 1 | 2 | 3 | 4 |
| 3. 我曾因為朋友或同事尖銳地批判我而感到不快。 | 0 | 1 | 2 | 3 | 4 |
| 4. 我肯定他人在背後嘲笑我。 | 0 | 1 | 2 | 3 | 4 |
| 5. 我一直在想人們怎麼避開我。 | 0 | 1 | 2 | 3 | 4 |
| 6. 有人一直在給我暗示和線索。 | 0 | 1 | 2 | 3 | 4 |
| 7. 我認為有些人表裏不一。 | 0 | 1 | 2 | 3 | 4 |
| 8. 人們在背後談論我使我感到不安。 | 0 | 1 | 2 | 3 | 4 |
| Part B |  |  |  |  |  |
| 1. 有些人對我有不好的意圖。 | 0 | 1 | 2 | 3 | 4 |
| 2. 因人們想我感到受威脅，所以他們盯著我。 | 0 | 1 | 2 | 3 | 4 |
| 3. 我肯定某些人曾做事來煩擾我。 | 0 | 1 | 2 | 3 | 4 |
| 4. 我相信曾有針對我的陰謀存在過。 | 0 | 1 | 2 | 3 | 4 |
| 5. 我肯定有人想傷害我。 | 0 | 1 | 2 | 3 | 4 |
| 6. 我不能停止想起別人怎樣嘗試迷惑我。 | 0 | 1 | 2 | 3 | 4 |
| 7. 我曾因被逼害而感到苦惱。 | 0 | 1 | 2 | 3 | 4 |
| 8. 很難不去想起他人怎樣要我難受。 | 0 | 1 | 2 | 3 | 4 |
| 9. 他人有意圖地對我懷敵意。 | 0 | 1 | 2 | 3 | 4 |
| 10. 我為有人想傷害我而感到憤怒。 | 0 | 1 | 2 | 3 | 4 |

**German Version of the R-GPTS**

**Revised Green et al. Paranoid Thoughts Scale (R-GPTS)**

**Deutsche Übersetzung**

Bitte lesen Sie sich jede der folgenden Aussagen sorgfältig durch. Die Aussagen beziehen sich jeweils auf Gedanken und Gefühle über andere, die Sie **während des letzten Monats** erlebt haben könnten. Bitte denken Sie an den letzten Monat zurück und geben Sie auf einer Skala von 0 (überhaupt nicht) bis 4 (völlig) an, **in welchem Ausmaß Sie diese Gedanken und Gefühle erlebt haben**.

(Bitte beziehen Sie Ihre Einschätzungen nicht auf Erlebnisse, die Sie unter Drogeneinfluss gemacht haben.)

|  | Überhaupt nicht |  |  |  | völlig |
| --- | --- | --- | --- | --- | --- |
| Part A |  |  |  |  |  |
| 1. Ich habe Zeit damit verbracht darüber nachzudenken, ob Freunde über mich lästern. | 0 | 1 | 2 | 3 | 4 |
| 2. Ich habe oft mitbekommen, dass Menschen über mich geredet haben. | 0 | 1 | 2 | 3 | 4 |
| 3. Es hat mich belastet, wenn Freunde und Kollegen mich kritisiert haben. | 0 | 1 | 2 | 3 | 4 |
| 4. Menschen haben mit Sicherheit hinter meinem Rücken über mich gelacht. | 0 | 1 | 2 | 3 | 4 |
| 5. Ich habe viel darüber nachgedacht, ob Menschen den Kontakt zu mir vermeiden | 0 | 1 | 2 | 3 | 4 |
| 6. Menschen haben mir gegenüber Andeutungen gemacht. | 0 | 1 | 2 | 3 | 4 |
| 7. Ich glaubte, dass bestimmte Leute nicht diejenigen waren, als die sie erschienen. | 0 | 1 | 2 | 3 | 4 |
| 8. Dass Menschen hinter meinem Rücken über mich reden, hat mich belastet. | 0 | 1 | 2 | 3 | 4 |
| Part B |  |  |  |  |  |
| 1. Bestimmte Personen hatten es auf mich abgesehen. | 0 | 1 | 2 | 3 | 4 |
| 2. Menschen starrten mich an, damit ich mich bedroht fühlte. | 0 | 1 | 2 | 3 | 4 |
| 3. Ich war mir sicher, dass bestimmte Personen Dinge getan haben, um mich zu nerven. | 0 | 1 | 2 | 3 | 4 |
| 4. Ich war davon überzeugt, dass es eine Verschwörung gegen mich gab. | 0 | 1 | 2 | 3 | 4 |
| 5. Ich war mir sicher, dass mir jemand Leid zufügen wollte. | 0 | 1 | 2 | 3 | 4 |
| 6. Ich konnte nicht aufhören, daran zu denken, dass Menschen mich verwirren wollen. | 0 | 1 | 2 | 3 | 4 |
| 7. Es hat mich belastet, verfolgt zu werden. | 0 | 1 | 2 | 3 | 4 |
| 8. Es war schwierig, nicht daran zu denken, dass Menschen wollten, dass es mir schlecht geht. | 0 | 1 | 2 | 3 | 4 |
| 9. Menschen waren mir gegenüber absichtlich feindselig. | 0 | 1 | 2 | 3 | 4 |
| 10. Ich war wütend darüber, dass mir jemand Leid zufügen wollte. | 0 | 1 | 2 | 3 | 4 |
